# Fabrication of impedimetric sensor based on metallic nanoparticle for the determination of mesna anticancer drug

**DOI:** 10.1038/s41598-023-38643-3

**Published:** 2023-07-14

**Authors:** Maryam Mehrban, Tayyebeh Madrakian, Abbas Afkhami, Nahid Rezvani Jalal

**Affiliations:** Faculty of Chemistry, Bu-Ali Sina University, Hamedan, 6517838695 Iran

**Keywords:** Pharmaceutics, Chemistry, Analytical chemistry, Electrochemistry

## Abstract

Electrochemical impedance spectroscopy (EIS) is a highly effective technique for studying the surface of electrodes in great detail. EIS-based electrochemical sensors have been widely reported, which measure the charge transfer resistance (R_ct_) of redox probes on electrode surfaces to monitor the binding of target molecules. One of the protective drugs against hemorrhagic cystitis caused by oxazaphosphorine chemotherapy drugs such as ifosfamide, cyclophosphamide and trophosphamide is Mesna (sodium salt of 2-mercaptoethanesulfonate). The increase in the use of Mesna due to the high consumption of anti-cancer drugs, the determination of this drug in biological samples is of particular importance. So far, no electrochemical method has been reported to measure Mesna. In this research, a novel impedimetric sensor based on a glassy carbon electrode (GCE) modified with oxidized multiwalled carbon nanotubes (MWCNTs)/gold nanoparticle (AuNPs) (denoted as Au NPs/MWCNTs/GCE) for impedimetric determination of Mesna anticancer drug was developed. The modified electrode materials were characterized by field emission scanning electron microscopy (FESEM), energy dispersive X-ray (EDX), and EIS. The electrochemical behavior of Mesna at the surface of Au NPs/MWCNTs/GCE was studied by an impedimetric method. The detection mechanism of Mesna using the proposed impedimetric sensor relied on the increase in the R_ct_ value of [Fe (CN)_6_]^3−/4−^ as an electrochemical probe in the presence of Mesna compared to the absence of Mesna as the analyte. Under the optimum condition, which covered two linear dynamic ranges from 0.06 nmol L^−1^ to 1.0 nmol L^−1^ and 1.0 nmol L^−1^ to 130.0 µmol L^−1^, respectively. The detection limit was 0.02 nmol L^−1^. Finally, the performance of the proposed sensor was investigated for Mesna electrochemical detection in biological samples.

## Introduction

Effective management of cancer requires optimizing the dose of anticancer drugs^1^. Mesna, the sodium salt of 2-mercaptoethanesulfonate, is commonly used to protect against hemorrhagic cystitis caused by oxazaphosphorine chemotherapy drugs like ifosfamide, cyclophosphamide, and trofosfamide. Mesna's free thiol group covalently binds to secondary metabolites of ifosfamide, preventing the formation of urotoxic acrolein. Acrolein binds to the bladder and kidney cells and causes cell death. Accurate determination of Mesna in biological samples is crucial due to its increasing use in cancer treatment and to minimize the side effects of the drug^2–4^. Therefore, it is essential to develop sensitive methods to measure Mesna levels in biological samples. Several methods, such as High-Performance Liquid Chromatography (HPLC)^5–7^, Raman scattering^8^, Spectrophotometry^9,10^, Fluorimetry^3,11^, and Argentometry^12^, have been proposed to estimate Mesna drug levels. However, these methods have some drawbacks, including low selectivity, long sample pretreatment, and extended analysis time. Electrochemical impedance spectroscopy (EIS) is a powerful technique widely used in analytical chemistry for designing electrochemical sensors. EIS is a label-free technique that can be used to study changes in the electrode surface, making it an ideal method for designing impedimetric sensors^13^. However, the electrode surface degrades after being used in the sample and needs to be modified before each determination.^14^. Until now, no electrochemical methods have been reported for determining Mesna levels, as per previous reports. Developing an electrochemical method for Mesna detection is necessary to overcome the limitations of the existing methods. In this technique, a sinusoidal potential wave is applied to a three-electrode system, and EIS data such as the real part of the impedance (Z_real)_, the imaginary part of the impedance (Z_imaginary_), and phase shift (ϕ) at different frequencies are recorded. The obtained Nyquist plot, which plots Z_imaginary_ against Z_real_, can be used to describe the electrical circuit characteristics, the characteristics of the electrode surface, and the analytical responses in the impedimetric sensor^15^. EIS can categorize into two groups: Faradaic and non-Faradaic. In the non-Faradic EIS, impedance response is based on DC potential and a double-layer capacitor. In contrast, in Faradaic EIS, the impedance response originates from the redox reaction^16^. Therefore, Faradaic EIS can be utilized for quantitative analysis^17^.

The unique properties of carbon nanotubes (CNTs), such as their high conductivity and stability, make them an excellent modification of electrochemical sensors. The simple mass manufacturing, low cost per unit, and improved thermal and chemical stability have made multi-walled CNTs (MWCNTs) more prominent than single walled CNTs (SWCNTs) and zigzag structures. Electrical and mechanical properties of SWCNTs can be altered when functionalized due to structural flaws causes by C=C bond break during chemical reaction. The intrinsic features of carbon nanotubes can, however, be preserved by chemically modifying MWCNTs’ outside wall. A further advantage of MWCNTs over SWCNTs is their ease of dispersion, which make them a good choice for composites, printing, and coating. Thus, MWCNTs are widely used to improve the properties of electrochemical sensors^18–21^.

Noble metal nanoparticles possess unique electrical and chemical properties, making them highly desirable materials for fabricating label-free sensors. Since gold nanoparticles (Au NPs) transfer free charge, they influence electron transfer kinetics, which enhances conductivity, catalytic and photocatalytic activity. In addition, as part of their characteristic interaction, Au NPs are capable of reacting with thiol-containing compounds. consequently, gold nanoparticle can also be used for sensors because of their affinity for bonds with thiols^22–24^.

**Figure 1:**
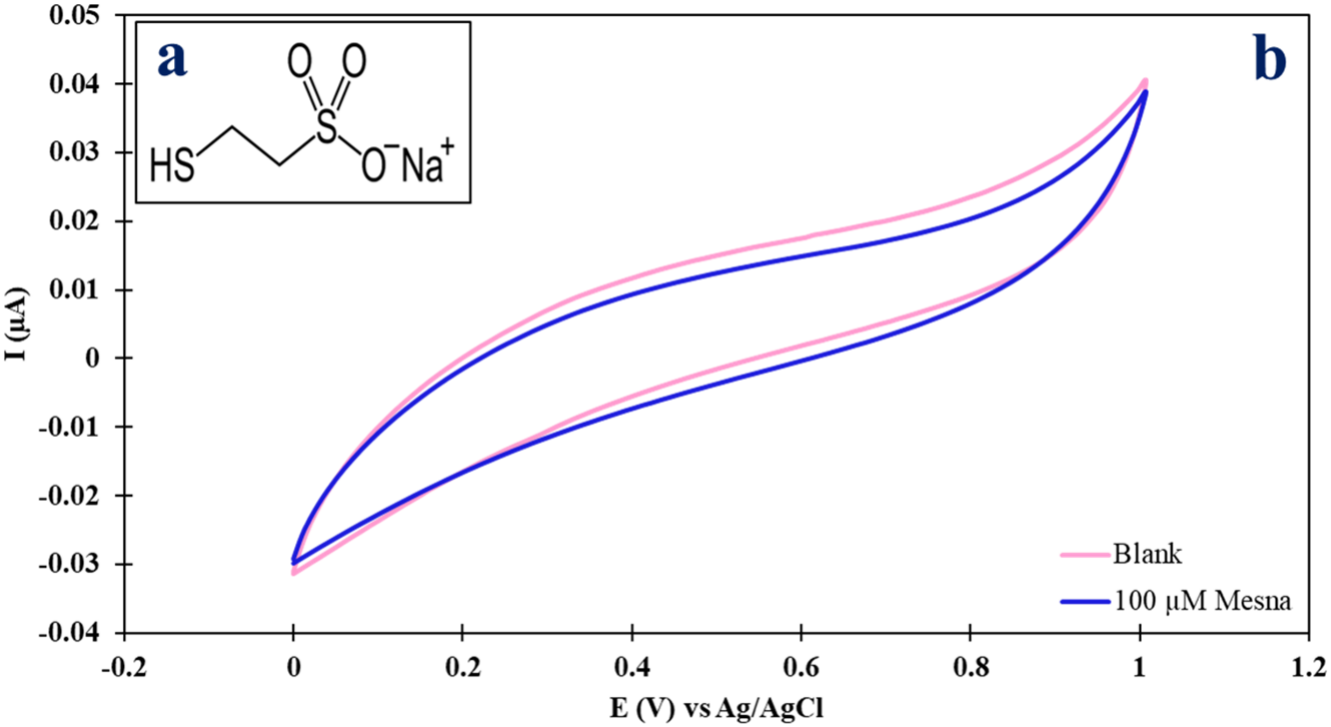
(**a**) Structure of Mesna. (**b**) CV curves of 100 µmol L^−1^ Mesna at bare GCE in B-R buffer solution (pH = 7.4) at a scan rate of 0.1 mV s^−1^.

This study presents a novel approach for the sensitive and selective determination of Mesna using an impedimetric sensor based on a glassy carbon electrode modified with AuNPs and MWCNTs. Figure 1a shows the Structure of Mesna and Fig. 1b demonstrates a cyclic voltammogram of 100 µmol L^−1^ Mesna in Britton–Robinson (B–R) buffer solution with the pH of biological fluids on the bare glassy carbon electrode. According to Fig. 1b, no visible anodic or cathodic peak was seen in the potential window of 0 to + 1 V vs. Ag/AgCl for the Mesna anticancer drug, The absence of visible anodic or cathodic peaks in the cyclic voltammogram of Mesna suggests that it is not active in the potential window of 0 to + 1 V vs. Ag/AgCl. Therefore, an indirect method was developed to detect Mesna by measuring the changes in the charge transfer resistance (R_ct_) of [Fe(CN)_6_]^3−/4−^ in the presence of Mesna compared to its absence. The use of MWCNTs and AuNPs as modifiers enhance the electron transfer kinetics on the electrode surface and increase the sensitivity of the sensor. The sensor was successfully applied for the determination of Mesna in serum and urine samples. Overall, this study offers a promising approach for the sensitive and selective detection of Mesna, which can contribute to the development of efficient and reliable anticancer drug management.

## Results and discussion

### Characterization of AuNPs/MWCNT/GCE

**Figure 2:**
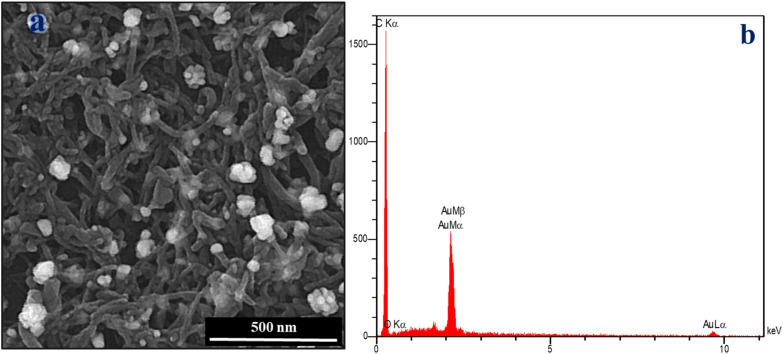
(**a**) FESEM image and (**b**) EDX analysis of AuNPs/MWCNT/GCE.

The surface morphology of AuNPs/MWCNTs/GCE was analyzed using field-emission scanning electron microscopy (FESEM) images. Figure 2a shows the FESEM image of AuNPs/MWCNTs/GCE. As shown in Fig. 2a, the intertwined tubular structure and aggregation of MWCNTs were observed to be dispersed on the surface of the GCE. Additionally, the spherical-shaped Au NPs were found to be homogeneously distributed over the surface of the MWCNTs/GCE. According to Fig. 2a, the average size of Au NPs was estimated to be about 32 nm. Additionally, elemental analysis of the modified electrode was conducted using EDX analysis. As Fig. 2b shows, the EDX spectrum of the AuNPs/MWCNTs/GCE indicates the presence of only three elements, namely C, Au, and O, with weight percentages of 71.3%, 27.1%, and 1.5%, respectively. These results demonstrate the successful fabrication of the AuNPs/MWCNTs/GCE.

**Figure 3:**
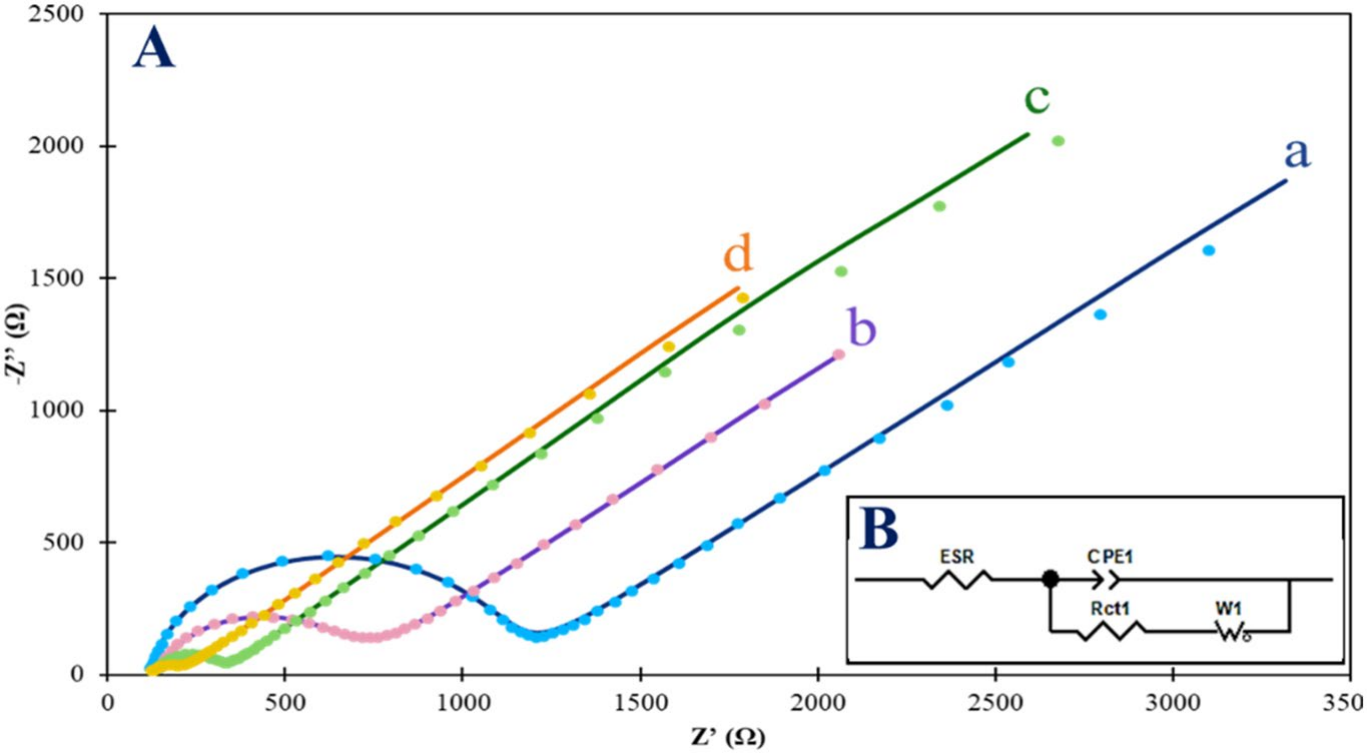
(**A**) Nyquist plots of the step-by-step modification of GCE in 0.1 mol L^−1^ KCl and 5 mmol L^−1^ of [Fe (CN)_6_]^3−/4−^: (a) bare GCE; (b) MWCNTs/GCE; (c) Au NPs/GCE and (d) Au NPs/MWCNTs/GCE at + 0.22 V from 0.01 Hz to 80 kHz. (**B**) Equivalent circuit of the step-by-step modification of GCE.

EIS was employed to characterize the step-by-step resistance of the Au NPs/MWCNTs/GCE. Theexperimental EIS data (dotted) and fitted EIS data (solid line) related to bare GCE (a), MWCNTs/GCE (b), Au NPs/ GCE (c), and Au NPs/MWCNTs/GCE (d) in the solution including 0.1 mol L^−1^ of KCl, 5 mmol L^−1^ of [Fe(CN)_6_]^3−/4−^ are shown in Fig. 3a. The EIS data were recorded in the potential of 0.22 V and frequency ranging from 0.01 HZ to 80 kHz. Therefore, the charge transfer resistance (R_ct_) value for the step-by-step electrode modification can be calculated by using the Nyquist plot^25^. The value of R_ct_ was estimated to be 992 Ω at the bare GCE (Fig. 3A, curve a). Following the modification of bare GCE via MWCNTs, the R_ct_ value decreased to 545 Ω for the redox probe (Fig. 3A, curve b). This result confirms that the presence of the MWCNTs layer facilitated the electron transfer between [Fe(CN)_6_]^3−/4−^ and the MWCNT-coated electrode^26^. As shown in Fig. 3A, curve c, The R_ct_ value of Au NPs/GCE decreased to 179 Ω, indicating that the presence of Au NPs facilitated the electron transfer from the redox probe to the surface of the electrode. On the Au NPs/MWCNTs/GCE, the R_ct_ value decreased significantly to 84 Ω. (Fig. 3A, curve d). This observation can be attributed to the synergistic effect of both MWCNT and Au NPs on the modified electrode, indicating the enhanced electrocatalytic activity of the Au NPs/MWCNTs/GCE. These results demonstrate that the bare electrode was successfully modified by Au NPs and MWCNTs.

The Randles–Ershler equivalent circuit was modeled for extracting the circuit parameters^27^. The equivalent circuit used to fit the experimental EIS spectrum is shown in Fig. 3B. The equivalent circuit used in this study comprises several elements, including the electrolyte resistance (ESR), the charge transfer resistance (R_ct_), a constant phase element (CPE) in place of the double layer capacitor, and the Warburg impedance (W)^28^. Moreover, the apparent charge transfer rate constant (k_app_) of the [Fe(CN)_6_]^3−/4−^ redox probe was calculated using Eq. 1^29^:

1 $${k}_{app}=\frac{R\times T}{{n}^{2}\times {F}^{2}\times A\times {C}^{*}\times {R}_{ct}}$$

where R is the ideal-gas constant, T treats the temperature in Kelvin, F is Faraday’s constant, n is the number of electron transfers, A is the surface-area electrode (hereon: 0.0314 cm^2^), and C^*^ is the concentration of [Fe(CN)_6_]^3−/4−^ (5 mmol L^−1^). Table 1 illustrated the more detailed data on EIS analysis obtained from spectra of Fig. 3 related to step-by-step electrode modification.

**Table 1:** The EIS parameters extracted from the spectra of Fig. 3a were obtained in different modification steps of GCE.

Electrode	ESR (Ω)	R_ct_ (Ω)	g^a^	C_dl_^b^ (μF)	k_app_ (cm s^−1^)
GCE	120	992	0.91	0.93	1.70 × 10^−3^
MWCNTs/GCE	139	545	0.82	3.37	3.10 × 10^−3^
Au NPs/GCE	133	179	0.85	1.54	9.44 × 10^−3^
Au NPs/MWCNTs/GCE	118	84	0.76	8.30	2.01 × 10^−2^

^a^The constant phase element as a circuit element was obtained from: Z_CPE_ = 1/Q(j ω)g, where Q is the factor of proportionality, j is an imaginary number, g is CPE exponent (− 1 < g < 1), and ω = 2πf is the angular frequency. In the case g =  − 1, CPE represents a pure inductor; g = 0, CPE is equivalent to a pure resistor; g = 0.5, CPE denotes diffusion behavior, and g = 1 indicates CPE is equivalent to a pure capacitor.

^b^Double layer capacitance.

According to Table 1, The k_app_ value for the AuNPs/MWCNTs/GCE was calculated as 2.01 × 10^–3^ cm s^−1^, which was found to be 11.8 times higher than that of the bare electrode (1.7 × 10^–3^ cm s^−1^). This indicates that electron transfer kinetics is significantly increased at the AuNPs/MWCNTs/GCE compared to the bare electrode. Additionally, the C_dl_ value, which is a measure of the charge on the surface of the AuNPs/MWCNT/GCE, was calculated as 8.3 μF, which is 2.46 and 5.38 times higher than that at the MWCNTs/GCE (3.37 μF) and AuNPs/GCE (1.54 μF) surfaces, respectively. The increase in the double-layer capacitance is attributed to the higher surface area due to the additional layer of modified electrode^30^. These results demonstrate that the proposed electrode exhibits an increase in electrochemical activity due to the increase in surface charge.

### Surface area study

**Figure 4:**
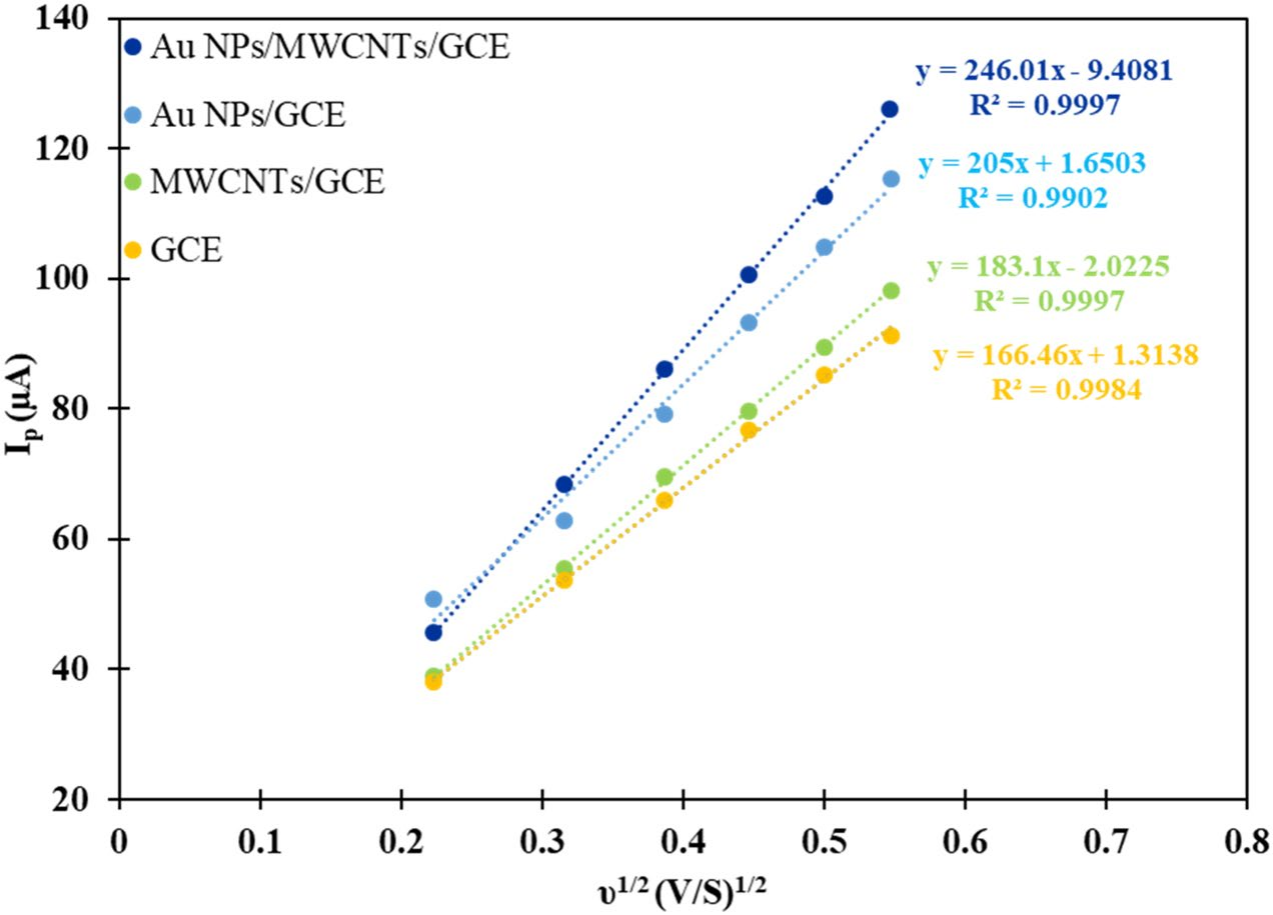
Plot of anodic peak current (µA) vs square root of scan rate (V/s)^1/2^ for electrode after each modification step in 0.5 mol L^−1^ KCl and 5 mmol L^−1^ of [Fe(CN)_6_]^3−/4−^.

The effective surface area of the bare GCE, MWCNTs/GCE, Au NPs/ GCE, and Au NPs/MWCNTs/GCE were obtained by cyclic voltammetry for 0.5 mol L^−1^ KCl electrolyte solution containing 5 mmol L^−1^ [Fe(CN)_6_]^3−/4−^ as a redox probe at different scan rates in the range of 5–300 mV. Randles–Sevcik Equation (Eq. 2) was used to estimate the effective surface area of the electrodes after each modification step and demonstrated in Table 2.

2 $${I}_{p}=2.69\times {10}^{5}{n}^\frac{3}{2}A{C}^{*}\sqrt{D\upsilon }$$

where I_p_ is the peak current, n is the number of electrons involved in the redox process, A is the surface area of the working electrode, D is the diffusion coefficient of the electroactive species, C^*^ is the bulk concentration of the electroactive species and υ is the scan rate. As can be seen from obtained results in Fig. 4, the modified electrode exhibits an increase in effective surface area due to the increase in the slope of the anodic peak current vs ν^1/2^ in different stages of surface modification^17^.

**Table 2:** Comparing the efficiency of the electrodes in different stages of surface modification along and the calculated values of the active surface of the electrodes.

Electrode	Surface area (mm^2^)	Slope (µA/V^1/2^ S^−1/2^)	Correlation coefficient (R^2^)
GCE	4.56	166.46	0.9984
MWCNTs/GCE	5.04	183.1	0.9997
Au NPs/GCE	5.64	205.6	0.9902
Au NPs/MWCNTs/GCE	6.70	246.01	0.9997

### Electrodeposition of Au NPs

**Figure 5:**
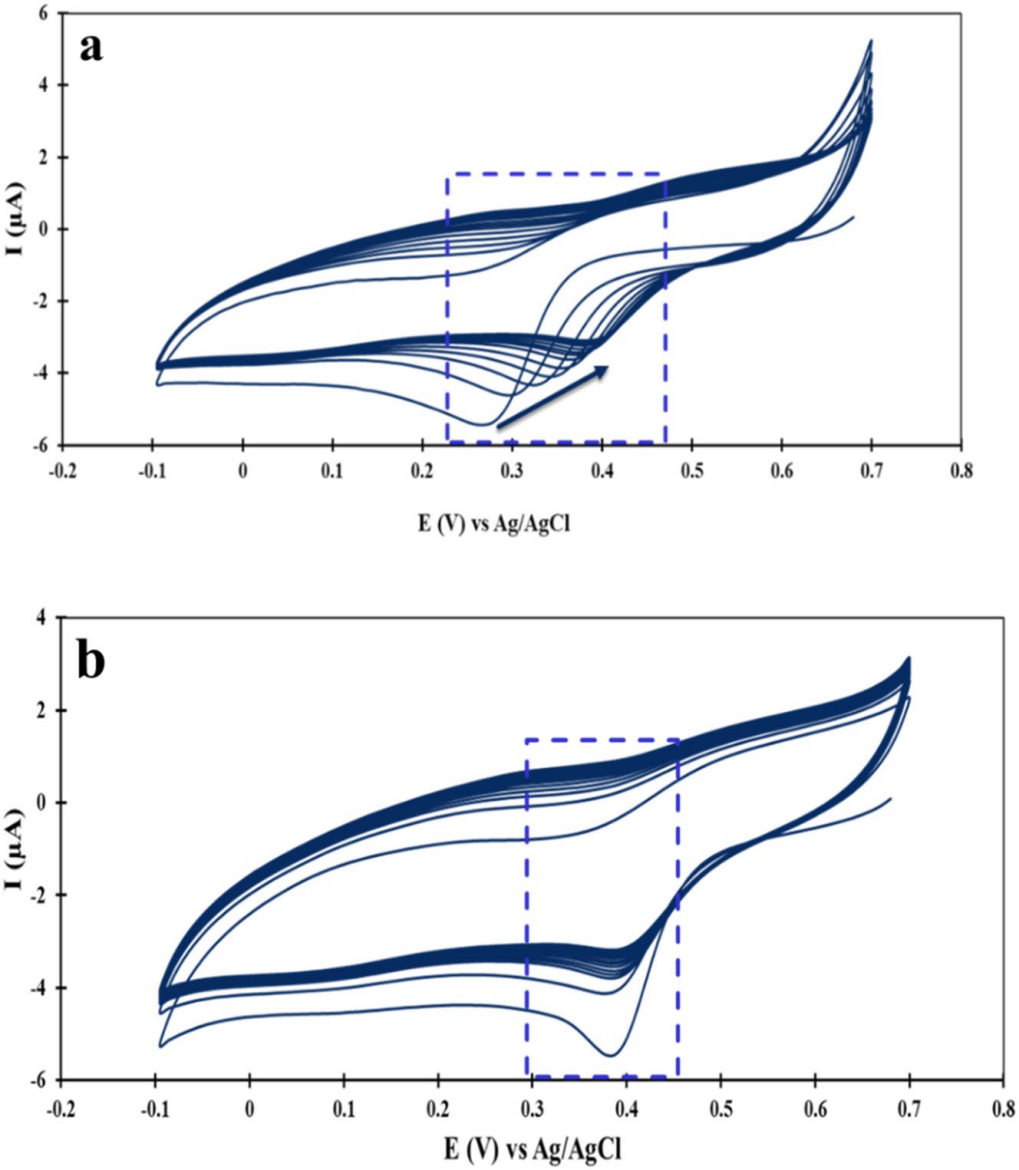
CV curves of electro-reduction Au(III) on the (**a**) bare GCE, and (**b**) MWCNTs/GCE in the potential range from + 0.7 to − 0.1 V at a scan rate of 50 mV s^−1^ in the solution containing KCl (0.1 mol L^−1^), HCl (2 mol L^−1^) and HAuCl_4_ (1 mmol L^−1^).

Figure 5a illustrates the electrodeposition of AuNPs on bare GCE by the CV technique. As shown in Fig. 5a, as the number of CV cycles increased, there was a decrease in the cathodic peak current, and the Epeak shifted toward lower negative potentials. This observation can be attributed to the dominant nucleation process of Au NPs on the GCE surface in the initial CV cycles. However, the growth of Au NPs becomes dominant in the subsequent CV cycles, making the electrodeposition easier^31^. The CV curves of the electro-reduction of Au(III) to precipitate Au(0) on MWCNTs/GCE in a 1 mmol L^−1^ HAuCl_4_ solution is shown in Fig. 5b. As Fig. 5b shows the cathodic peak potential at MWCNTs/GCE (Epeak = 0.38 V) shifted to a lower negative potential compared to the bare GCE. This observation is attributed to the conductivity of MWCNTs on the bare GCE, which facilitates electron transfer.

### Optimization of the Au NPs/MWCNT/GCE fabrication

To attain the best sensor efficiency and maximum sensitivity, the efficacy of impressive parameters was studied. Then, the analytical signal is calculated based on Eq. (3):

3 $$\Delta {R}_{ct}={R}_{ct }-{R}_{ct0}$$

where R_ct_ is the charge transfer resistance of [Fe(CN)_6_]^3−/4−^ after Mesna conjunct on the modified electrode and R_ct0_ is the charge transfer resistance of [Fe(CN)_6_]^3−/4−^ at Au NPs/MWCNTs/GCE as a blank signal.

**Figure 6:**
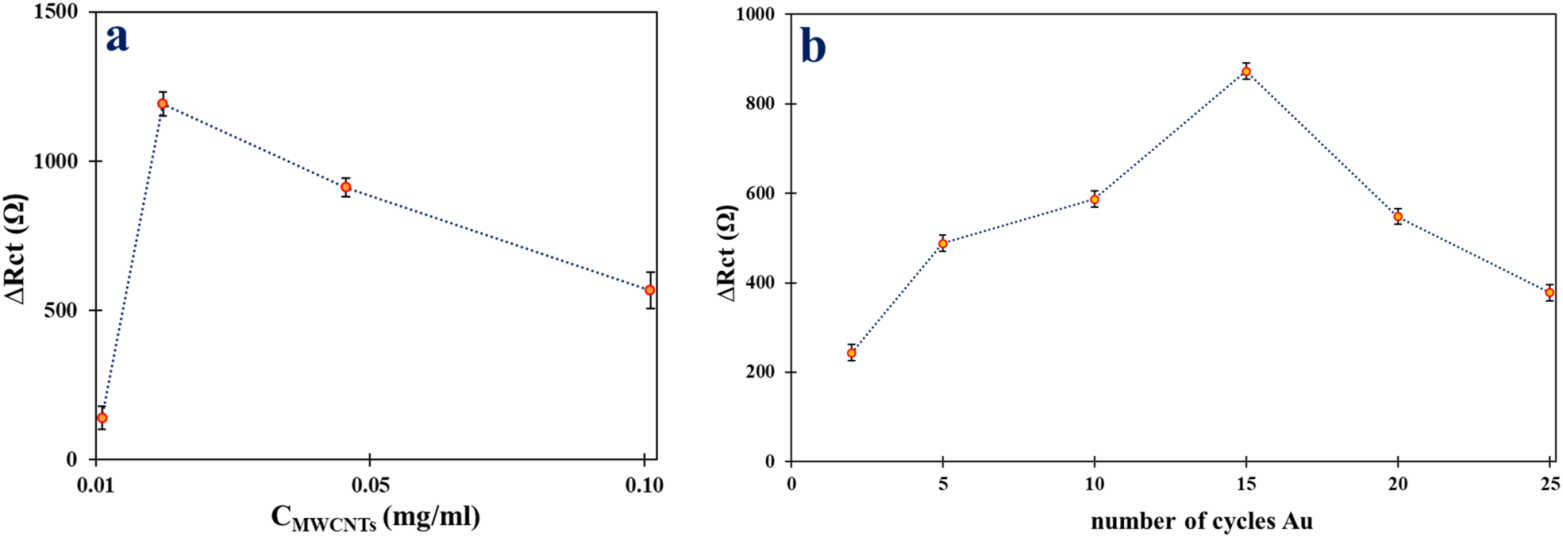
The effect of (**a**) concentration of MWCNTs and (**b**) the number of CV cycles of Au(III) electrodeposition on ΔR_ct_ onto Au NPs/MWCNTs/GCE.

The effect of cycle number for Au(III) electrodeposition and concentration of MWCNTs on the fabrication of the modified electrode was investigated. The changes in R_ct_ values (∆R_ct_) were studied for different MWCNTs concentrations (0.01, 0.02, 0.05, and 0.1 mg mL^−1^) and various numbers of cycles (2, 5, 10, 15, 20, and 25 cycles). The thickness of the cover layer was determined based on the concentration of MWCNTs, which was effective for the sensitivity of the detection method. Loading a specific amount of conductive MWCNTs enhanced the conductivity and chemical sensitivity of the electrode. Figure 6a shows that ∆R_ct_ augments by increasing the concentration of MWCNTs and overtakes a maximum in the attendance of 0.02 mg mL^−1^ of MWCNTs dispersal due to the increase in the active surface area of the electrode and then decreases. The decrease in ΔR_ct_ by increasing MWCNTs can be attributed to increasing the CNT-CNT contact resistance owing to the increasing thickness of the coated film. Thus, future analysis was performed in 0.02 mg mL^−1^ MWCNTs as the optimum concentration^32^.

Indirect determination of mesna based on a self-assembly interaction-free thiol group of Mesna with Au NPs on the modified electrode^33^. As shown in Fig. 6b, the ΔR_ct_ values increased as the number of cycles increased from 2 to 15, due to an increase in active sites for strong bonds between the SH group of Mesna and Au NPs on the modified electrode. The ΔR_ct_ values reached a maximum at 15 cycles, after which they began to decrease. This decrease may be attributed to the nucleation process stopping and the growth process starting for the Au NPs, leading to a decrease in active sites on the electrode surface^31^. Therefore, the 15 number of CV cycles for the reduction of Au(III) to Au(0) on MWCNTs/GCE was selected.

### Optimization of pH

**Figure 7:**
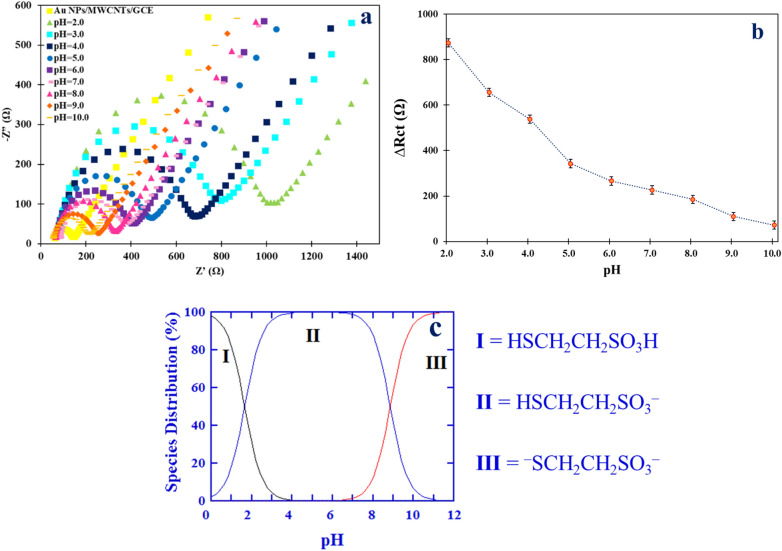
(**a**) Nyquist plots of [Fe(CN)_6_]^3−/4−^ recorded at Au NPs/MWCNTs/GCE in the presence of 30 nM Mesna in B–R buffer solution with different pH values (2.0–10.0). (**b**) Corresponding plot of ΔR_ct_ vs. pH. (**c**) Distribution diagram of species of Mesna vs. pH^33^.

The solution pH can affect both the analyte structure and the electrode surface. Therefore, the optimization of the supporting electrolyte pH is essential before studying the electrochemical behavior of Mesna. Direct electrochemical detection of Mesna has not been reported in the literature. Therefore, the basis of electrochemical detection of Mesna on the Au NPs/MWCNTs/GCE relied on indirect electrochemical detection. Thence, the change in the ΔR_ct_ value for the reaction of the redox probe as a function of Mesna concentration can be used as an analytical signal for the determination of Mesna. The presence of Mesna on the Au NPs/MWCNTs/GCE (i.e., Mesna/Au NPs/MWCNTs/GCE) leads to increases in ΔR_ct_ value compared to Au NPs/MWCNTs/GCE. Therefore, the interaction between Mesna and the surface of the modified electrode in the pH range of 2.0–10.0 using B-R buffer solution (0.04 mol L^−1^) was investigated by the open circuit voltage method, and then the Nyquist plots were recorded. As shown in Fig. 7a, The Nyquist plots of [Fe(CN)_6_]^3−/4−^ showed that the ΔR_ct_ value decreased as the pH value increased. The maximum ΔR_ct_ value was observed at pH 2.0, and then it gradually decreased as the pH increased (Fig. 7b). These results suggest that the presence of Mesna on the AuNP/MWCNT/GCE impedes the electron transfer from the redox probe to the surface of the electrode. As shown in Fig. 7c, these observations can be explained by the distribution diagram of Mesna species at different pH values. Based on the reported pK_a_ values of Mesna (pK_a1_ = 1.2–2.0, pK_a2_ = 8.8)^33^, at pH values greater than 8.8, the dominant form of the drug will carry two negative charges as the S–H group of the Mesna can lose its proton (i.e., ^−^SCH_2_CH_2_SO_3_^−^) and the drug is mainly present in the Dimesna form, which is not the target species for determination. At pH values between 8.8 and 2.2, Mesna is present in its negative form (i.e., HSCH_2_CH_2_SO_3_^−^) and can bind to the Au NPs on the electrode surface. Additionally, the dominant form of Mesna in this pH range is negatively charged and is repelled by the negatively charged MWCNTs due to the electrostatic repulsion between the negatively charged Mesna and the negatively charged MWCNTs (pH_PZC_ = 2.66)^34^ on the electrode surface. At pH 2.0, Mesna is mainly in its neutral form (HSCH_2_CH_2_SO_3_H) and MWCNTs become positively charged^35^. However, due to an acidic error in the pH adjustment of the B–R buffer solution, the interaction between Mesna and the surface of the modified electrode was not investigated in pH values below 2.0. Therefore, Mesna can interact with the Au NPs and carboxylic groups of MWCNTs through the S–H group and SO_3_H groups, respectively^36^. Consequently, further analysis was performed at pH 2.0. The interaction between Mesna and the surface of the modified electrode was investigated at pH values below 2.0, but reproducible responses were not obtained.

### Optimization of incubation time

**Figure 8:**
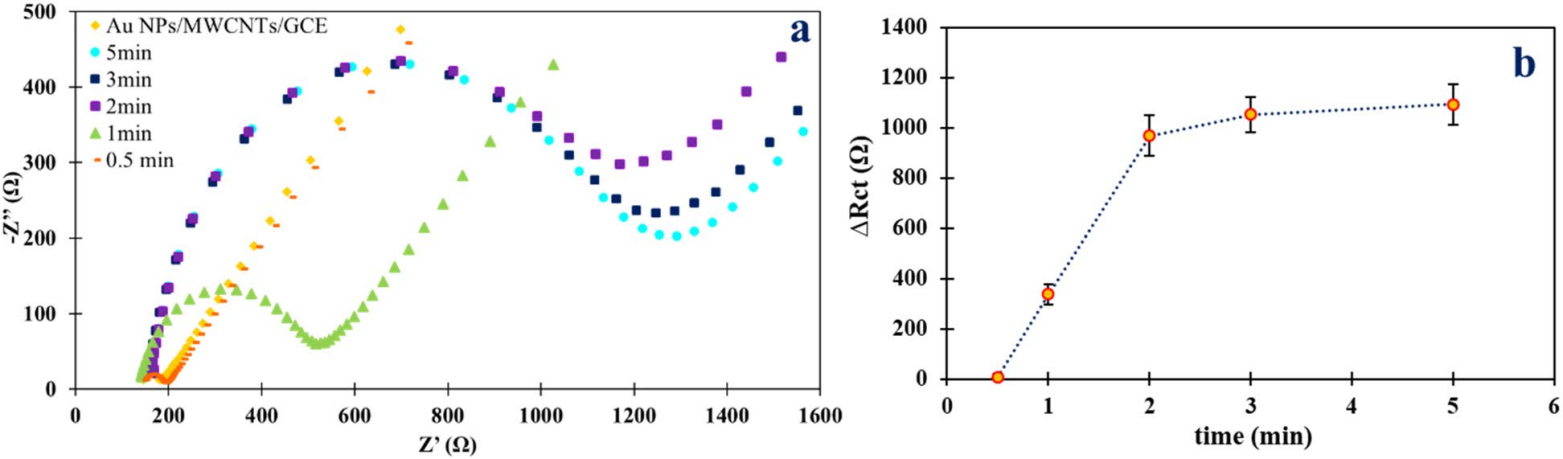
(**a**) Nyquist plots of [Fe(CN)_6_]^3−/4−^ recorded at Au NPs/MWCNTs/GCE in the presence of 30 nmol L^−1^ Mesna in B–R buffer solution with various incubation times. (**b**) Corresponding plot of ΔR_ct_ vs. time.

The incubation time for binding Mesna (30.0 nmol L^−1^) to the electrode surface was studied over a range of 0.5–5 min. The duration of incubation is a critical parameter that affects the interaction of Mesna with the electrode surface and, consequently, the sensitivity of the detection method. As shown in Fig. 8a,b, the ΔR_ct_ value increased by increasing the time up to 2 min and then plateaued with a further increase in the incubation time^37^. Therefore, an incubation time of 2 min was chosen as the optimum time.

### Analytical performance

**Figure 9:**
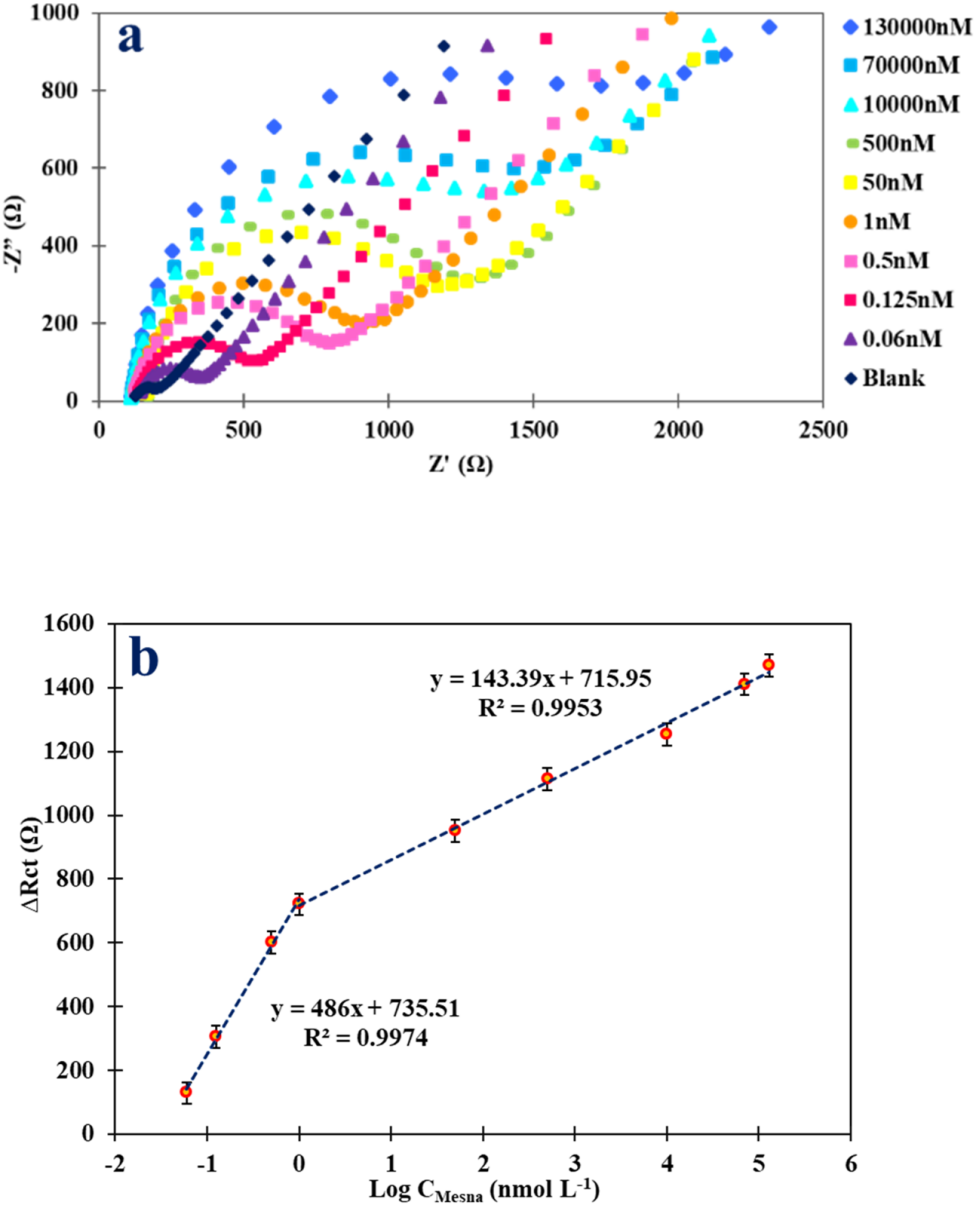
(**a**) Nyquist plots of [Fe(CN)_6_]^3−/4−^ recorded at Au NPs/MWCNTs/GCE in the different concentrations of Mesna in B–R buffer solution (pH = 2.0) with an incubation time of 2 min. (**b**) Corresponding plot of ΔR_ct_ vs. Log C_Mesna_.

The optimized conditions were used to evaluate the analytical performance of the proposed impedimetric sensor for various concentrations of Mesna. As shown in Fig. 9a, the EIS responses demonstrated an increase in the R_ct_ with increasing concentrations of Mesna. Two linear dynamic ranges (LDRs) for Mesna determination were obtained (Fig. 9b). The first linear dynamic range, ranging from 0.06 to 1.0 nmol L^−1^, has a slope of 486 Ω/nmol L^−1^ and an intercept of 731.51 Ω. The second linear dynamic range, ranging from 1.0 nmol L^−1^ to 130.0 µmol L^−1^, has a slope of 143.39 Ω/nmol L^−1^ and an intercept of 715.95 Ω. The correlation coefficients (R^2^) for the first and second LDR were 0.9974 and 0.9953, respectively. The first LDR is more sensitive, while the second LDR has a wider linear range. The self-assembly between Mesna and the electrode surface dominates at the first LDR and results in higher sensitivity. In other words, the number of active sites on the electrode surface is greater at lower concentrations of Mesna compared to higher concentrations of Mesna. Therefore, sensitivity decreases in the second LDR. Table 3 represents more detailed data on calibration curve analysis.

**Table 3:** Analytical parameters of the calibration curve for Mesna detection.

Regression equation	LDR (nmol L^−1^)	Slope (Ω/nmol L^−1^)	Correlation coefficient (R^2^)	DL (nmol L^−1^)
ΔR_ct_ (Ω) = 486 log C_Mesna_ + 735.51	0.06–1.0	486	0.9974	0.02
ΔR_ct_ (Ω) = 143.39 log C_Mesna_ + 715.95	1.0–130,000.0	143.39	0.9953	–

The detection limit (DL) is defined as the lowest concentration of the analyte that can be reliably detected by the analytical method based on the blank signal. In the present study, the DL was determined as 3 times the standard deviation of the blank sample divided by the slope of the calibration curve, which was found to be 0.02 nmol L^−1^. The obtained DL was lower than the acceptable concentration of Mesna in biological samples (i.e. 100 µmol L^−1^)^38^, indicating the high sensitivity of the proposed sensor for the detection of Mesna in real samples. Also, a comparison between the proposed sensor and other methods^3,5–11^ for the determination of Mesna is provided in Table 4. The linear range and limit of detection of the Au NPs/MWCNTs/GCE are better than those reported in previous methods. The presented impedimetric method has several notable features, including the synergistic effect of the electrocatalytic properties of Au NPs and MWCNTs, as well as the strong interaction between Mesna and the electrode surface, which enhances the sensitivity of the modified electrode.

**Table 4:** The comparison of the proposed modified electrode and others reported in the determination of Mesna.

Methods	LDR (µmol L^−1^)	DL (µmol L^−1^)	Reference
HPLC	0.16 × 10^–3^-30 × 10^–3^	0.04 × 10^–3^	^5^
HPLC	10–300	3.21	^6^
HPLC	2.5 × 10^–3^-2.5	1.64 × 10^–3^	^7^
Raman scattering	0.09–0.9	0.116 × 10^–3^	^8^
Spectrophotometry	6–60	0.92	^9^
Spectrophotometry	1.2–12.1	610 × 10^–3^	^10^
Fluorimetry	50–450	4.5	^11^
Argentometry	12–168	15.1	^12^
Fluorimetry	0.2–12	26 × 10^–3^	^3^
EIS	0.06 × 10^–3^–1.0 × 10^–3^ 1.0 × 10^–3^–130.0	0.02 × 10^–3^	This work

### Reproducibility and repeatability

**Figure 10:**
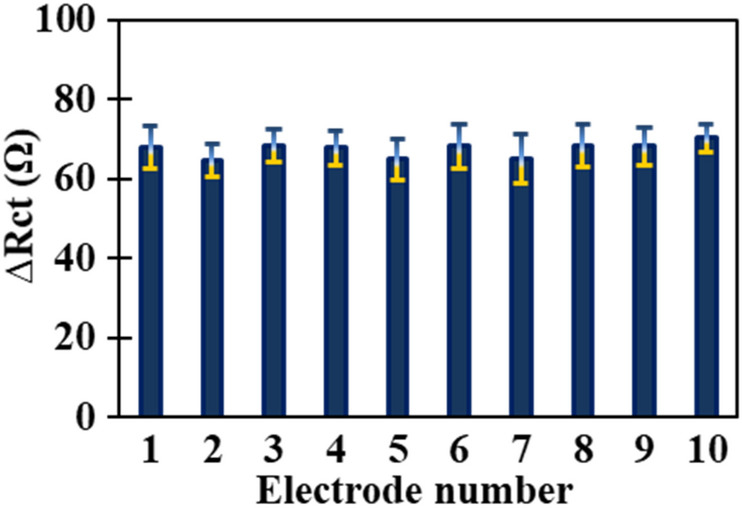
Repeatability of response at Au NPs/MWCNTs/GCE in 5 mmol L^−1^ of [Fe(CN)_6_]^3−/4−^.

The reproducibility of the Au NPs/MWCNTs/GCE was investigated using five parallel modified electrodes in a solution containing 1.0 nmol L^−1^ Mesna. The RSD% for the five parallel modified electrodes was found to be 4.6%. Since the thiol group of the drug interacts very strongly with the Au NPs, this results in the memory effect remaining on the electrode surface, which has both advantage and disadvantage. In the advantage, the Mesna drug interacts strongly with the electrode surface; in the disadvantage, the electrode surface must be polished and modified after each determination. Fortunately, the electrode surface can be modified in a simple and repeatable fashion, and it isn’t costly or time-consuming. To check the repeatability of the proposed electrode surface modification, 10 glassy carbon electrodes were modified with AuNPs and MWCNTs and the Nyquist plots of [Fe(CN)_6_]^3−/4−^ recorded at Au NPs/MWCNTs/GCE. According to Fig. 10, the RSD% for charge transfer resistance of [Fe(CN)_6_]^3−/4−^ at Au NPs/MWCNTs/GCE was found to be 2.7%. It is clear from these results that the fabrication of the Au NPs/MWCNTs/GCE has proper repeatability. Furthermore, the analytical signal is the ∆R_ct_ value , which is the difference between the charge transfer resistance from binding Mesna to modified electrode and the charge transfer resistance of Au NPs/MWCNTs/GCE as a blank signal. Due to this, there will be no problem in repeatability of the response in the measurements of Mesna.

### Evaluation of interferences

To evaluate the selectivity of the modified electrode, the ability of the proposed sensor to detect Mesna in the presence of some compounds commonly found in biological fluids was investigated (Table 5). The limit of tolerance was determined as the highest concentration of interfering substances that caused an error of less than 5% in the measurement of Mesna (1.0 nmol L^−1^). The results showed that the R_ct_ of [Fe(CN)_6_]^3−/4−^ for the determination of Mesna (1.0 nmol L^−1^) was only affected by the presence of L-cysteine. However, this issue can be resolved by pre-treating the real sample to precipitate proteins by adding acetonitrile^39^. Therefore, the presence of L-cysteine in real samples does not affect the EIS response. The results demonstrated that the determination of Mesna in real biological fluids was not affected by the presence of L-cysteine.

**Table 5:** Interference of some foreign species for detection of Mesna (1.0 nmol L^−1^).

Foreign species	Tolerable concentration (analyte/interfering species)
Na^+^, K^+^, NH_4_^+^, Mg^2+^, Cl^−^, Br^−^, NO_3_^−^, SO_4_^2−^,CO_3_^2−^	1:1000
Dopamine, Uric acid, Glucose	1:800
Ascorbic acid	1:700
Urea	1:600
Phenylalanine	1:200
l-cysteine	1:1

### Real sample analysis

To assess the feasibility of the proposed sensor for the analysis of the real samples, it was utilized for the detection of Mesna in urine and serum samples obtained from patient volunteers and healthy volunteers using the EIS method. The urine and serum samples of the health volunteers were found to be free from Mesna so synthetic samples were prepared by adding known amounts of Mesna to these samples. The results are given in Table 6. The recoveries were in the range of 94–103.2% which is satisfactory. The results of the analysis of patient volunteers are shown in Table 7. The proposed Au NPs/MWCNTs/GCE was able to detect Mesna in the real samples (urine and serum) without any pre-concentration step. Additionally, different concentrations of Mesna were spiked into the real samples and satisfactory recoveries in the range of 93.5% to 106.6% were obtained. Additionally, to validate the performance of the method, the standard addition method was employed and the value of Mesna in the real sample of a cancer patient volunteer was obtained as 1.7 ± 0.09 µmol L^−1^. These observations indicated that the proposed sensor can successfully assess the Mesna drug in the biological sample sans preparation. Therefore, Au NPs/MWCNTs/GCE can be suggested to determine Mesna in biological fluids.

**Table 6:** The obtained results of Mesna determination in real samples of healthy volunteers.

Sample	Added (µmol L^−1^)	Found (µmol L^−1^)	CV (%)	Recovery (%)
Plasma	4.0 × 10^–4^	(4.03 ± 0.21) × 10^–4^	5.4	100.7
	4.0 × 10^–2^	(4.13 ± 0.07) × 10^–2^	1.9	103.2
	90.0	86.54 ± 3.22	3.73	96.1
Urine	4.0 × 10^–4^	(3.76 ± 0.14) × 10^–4^	3.92	94
	4.0 × 10^–2^	(4.11 ± 0.1) × 10^–2^	2.5	102.7
	90.0	86.78 ± 0.43	0.5	96.4

**Table 7:** The obtained results of Mesna determination in real samples of patients volunteers.

Sample	Added (µmol L^−1^)		Found (µmol L^−1^)	CV (%)	Recovery (%)
Plasma	Volunteer 1	0	6.76 ± 0.17	2.6	–
		20.0	26.79 ± 0.34	1.3	100.1
		70.0	72.49 ± 0.23	0.32	93.9
	Volunteer 2	0	3.81 ± 0.26	7.0	–
		20.0	23.87 ± 0.1	0.45	100.3
		70.0	71.94 ± 0.55	0.77	97.3
	Volunteer 3	0	4.51 ± 0.06	1.5	–
		20.0	25.11 ± 0.24	0.98	103.0
		70.0	71.12 ± 0.45	0.64	95.1
Urine	Volunteer 1	0	1.02 ± 0.04	4.9	–
		20.0	21.37 ± 0.07	0.36	101.7
		70.0	68.02 ± 0.29	0.44	95.7
	Volunteer 2	0	1.24 ± 0.03	2.5	–
		20.0	19.95 ± 0.1	0.54	93.5
		70.0	72.44 ± 0.79	1.1	101.7
	Volunteer 3	0	1.20 ± 0.08	6.7	–
		20.0	20.89 ± 0.06	0.30	98.4
		70.0	75.85 ± 0.64	0.85	106.6

## Conclusion

This study presents a method to create an impedimetric sensor with excellent performance based on Au NPs and MWCNTs for the indirect detection of Mesna. To our best knowledge, there is no electrochemical determination of Mesna was reported still now. The fabrication of the modified electrode was confirmed with FESEM, EDX, and EIS methods, and the electrical conductivity and electrocatalytic ability of Au NPs and MWCNTs in the modified electrode allowed for high-sensitivity Mesna detection. The proposed method indirectly measured Mesna and achieved broad LDRs from 0.06 nmol L^−1^ to 1.0 nmol L^−1^ and 1.0 nmol L^−1^ to 130.0 µmol L^−1^ with a DL of 0.02 nmol L^−1^. This impedimetric sensor showed excellent sensitivity, extremely low detection limit, and wide linear range in comparison with some reported methods. The suggested impedimetric sensor exhibits a lower DL than the levels of Mesna in real urine and serum samples, which suggests a potential for the expansion of impedimetric sensors for the indirect detection of other analytes. This could provide a simple and suitable method for the quantitative determination of the nanomole level of Mesna for clinical laboratories. Although this method is the first electrochemical method reported for the determination of Mesna drug in an indirect way, the used electrode cannot be regenerated after interacting with Mesna and the electrode surface should be modified for each measurement.

## Materials and methods

### Chemicals and reagents

All chemicals and reagents were obtained from Merck Company (Darmstadt, Germany) or Fluka Company (Switzerland) and used without further purification. Multi-walled carbon nanotubes (MWCNTs) with lengths of 30 nm, diameter of 10–20 nm, and purity of 95% were obtained from Neutrino Company (Tehran, Iran). Mesna was purchased from VarianPharmed Pharmaceutical Company (Tehran, Iran). Britton-Robinson (B-R) buffers were prepared from 0.04 mol L^−1^ boric acid, 0.04 mol L^−1^ acetic acid, and 0.04 mol L^−1^ phosphoric acid. Also, NaOH (0.5 mol L^−1^) and HCl (0.5 mol L^−1^) were used to regulate the solution pH. A Mesna stock solution (5 mmol L^−1^) was prepared in deionezied water (DIW). Working solutions of Mesna were prepared daily by diluting the stock solution with the B–R universal buffer. DIW was used to prepare all solutions. All measurements were performed at ambient temperature.

### Instrumentation

A three-electrode system was employed, which included a working electrode (either bare GCE or modified GCE), a Pt rod electrode as the counter electrode, and an Ag/AgCl commercial electrode (3.0 mol L^−1^ KCl) as the reference electrode. EIS was utilized to study the electrochemical process of 5 mmol L^−1^ [Fe(CN)_6_]^3−/4−^ as a probe solution in 0.1 mol L^−1^ KCl. CV and EIS were performed using an Autolab potentiostat/galvanostat, Model PGSTAT 302 N (Ecochemie, Netherlands), and were controlled by a computer using Nova version 1.11 software. The experimental EIS data was analyzed using Z-View software (version 3.1c, Scribner Associates Inc, NC). A pH meter Model 827 (Metrohm, Switzerland) was used for pH measurements. In addition, experiments utilized Sartorius scales and a Hitachi centrifuge. The structural morphology was conducted using field-emission scanning electron microscopy and the elemental analysis was perused using energy-dispersive X-ray spectroscopy (MIRA3 TESCAN-XMU, Kohoutovice, Czech Republic).

### Preparation of the activated MWCNTs

The activated MWCNTs were obtained according to a previously reported procedure^18^. To oxidize the surface of the MWCNTs, 1 g of pristine MWCNTs were refluxed in H_2_SO_4_:HNO_3_ (3:1 v/v) under severe stirring at 130 °C for 30 min, and then neutralized by washing with DIW. The activated MWCNTs (referred to as MWCNTs) were then washed with DIW and ethanol and dried in an oven at 80 °C for 24 h. Subsequently, 1 mg of MWCNTs was dispersed in 2 mL of DIW using an ultrasonic device and kept for further use.

### Fabrication of the Au NPs/MWCNTs/GCE

The bare GCE was polished using alumina slurry on a polishing pad for approximately 5 min, and then sonicated in a solution containing DIW and ethanol (3:1 v/v) for 10 min. The GCE surface was activated by cycling the potential from + 0.3 to + 1.5 vs. Ag/AgCl at a scan rate of 0.1 mV s^−1^ in 1 µmol L^−1^ H_2_SO_4_ for 20 cycles. Subsequently, the GCE surface was washed with DIW. A thin film of MWCNTs was formed on the GCE surface by dropping 5 µL of MWCNTs on it and allowing it to dry at ambient temperature (MWCNT/GCE). Then, Au NPs were synthesized on MWCNTs/GCE by cycling the potential from 0.7 V to − 0.1 V vs. Ag/AgCl at a scan rate of 50 mV s^−1^ for 15 cycles in a solution containing 0.1 mol L^−1^ KCl, 2 mol L^−1^ HCl, and 1 mmol L^−1^ HAuCl_4_ (Au NPs/MWCNT/GCE)^23^.

### Preparation of the real sample

Serum and urine samples were collected from cancer patients and healthy volunteers who were laboratory personnel. Before analysis with the proposed sensor, the biological samples were analyzed without spiking and then after spiking with known amounts of Mesna. To prepare the serum samples, 1 mL of acetonitrile was added to 1 mL of serum to precipitate serum protein, followed by centrifugation for 10 min at 4000 rpm. The supernatant was carefully collected and diluted with a B-R buffer solution to reach an optimum pH of 2.0^24^. Urine samples were also prepared by centrifuging at 4000 rpm for 15 min, followed by filtration with a Whatman 42 filter paper to obtain a clear solution, which was then diluted with a B-R buffer solution at the optimum pH of 2.0^18^.

### Ethics approval

The study was approved by the ethics committee of the Hamedan University of Medical Sciences (Bessat Hospital) in Hamedan, Iran. All subjects gave written informed consent and all methods were carried out following the Declaration of Helsinki.

### Consent to participate

Informed consent was obtained from all individual participants included in the study.

## Data Availability

Experimental data will be available on request, please contact the corresponding author at madrakian@basu.ac.ir.

## References

[1] Paci A, Veal G, Bardin C, Levêque D, Widmer N, Beijnen J, Astier A, Chatelut E (2014). Review of therapeutic drug monitoring of anticancer drugs part 1–cytotoxics. Eur. J. Cancer.

[2] Rizk M, Anwer Taha E, Mowaka S, Mosaad Abdallah Y (2013). Kinetic fluorimetric determination of Mesna (Sodium-2-mercaptoethane sulfonate) in drug products through oxidation with cerium (IV). Eur. J. Chem..

[3] Hu B, Zhu J, Shen J, Yang L, Jiang C (2022). A portable sensing platform using an upconversion-based nanosensor for visual quantitative monitoring of mesna. Anal. Chem..

[4] Cutler, M. J. Pharmacokinetics and therapeutic uses of mesna. The University of Western Ontario (Canada) (2010).

[5] Głowacki R, Wójcik K, Bald E (2001). Facile and sensitive method for the determination of mesna in plasma by high-performance liquid Chromatography with ultraviolet detection. J. Chromatogr. A.

[6] Verschraagen M, Bosma M, Zwiers T, Torun E, Vijgh W (2003). Quantification of mesna and total mesna in kidney tissue by high-performance liquid Chromatography with electrochemical detection. J. Chromatogr. B.

[7] Mare S, Penugonda S, Ercal N (2005). High performance liquid chromatography analysis of MESNA (2-mercaptoethane sulfonate) in biological samples using fluorescence detection. Biomed. Chromatogr..

[8] Zheng X, Chen Y, Bi N, Qi H, Chen Y, Wang X, Zhang H, Tian Y (2011). Determination of the sodium 2-mercaptoethanesulfonate based on surface-enhanced Raman scattering. Spectrochim. Acta Part A Mol. Biomol. Spectrosc..

[9] Rizk M, Taha E, Mowaka S, Abdallah Y (2012). Kinetic spectrophotometric determination of mesna in drug substance and drug product using alkaline potassium permanganate. Chem. Sci. Rev. Lett..

[10] Ahmed N, Sattam Hamed Z, Kalaf M (2018). Determination of mesna in pharmaceutical preparations and environmental samples: Application to content uniformity testing. Int. J. Enhanc. Res. Sci. Technol. Eng..

[11] Su J, Feng Ch, Wu Y, Liang J (2019). A novel gold-nanocluster-based fluorescent sensor for detection of sodium 2-mercaptoethanesulfonate. RSC Adv..

[12] Ahmed N (2020). Application of argent metric in the estimation of mesna in tablets and injections: Application to content uniformity testing. J. Infect. Dis. Case Rep..

[13] Hu Y, Zuo P, Ye B (2013). Label-free electrochemical impedance spectroscopy biosensor for direct detection of cancer cells based on the interaction between carbohydrate and lectin. Biosens. Bioelectron..

[14] Mahmoud A, Tang T, Harrison D, Lee W, Jemere A (2014). A regenerating self-assembled gold nanoparticle-containing electrochemical impedance sensor. Biosens. Bioelectron..

[15] Magar H, Hassan R, Mulchandani A (2021). Electrochemical impedance spectroscopy (EIS): Principles, construction, and biosensing applications. Sensors.

[16] Faria R, Heneine L, Matencio T, Messaddeq Y (2019). Faradaic and non-faradaic electrochemical impedance spectroscopy as transduction techniques for sensing applications. Int. J. Biosens. Bioelectron.

[17] Soltani-Shahrivar M, Afkhami A, Madrakian T, Rezavani Jalal N (2023). Sensitive and selective impedimetric determination of TNT using RSM-CCD optimization. Talanta.

[18] Ghapanvari M, Madrakian T, Afkhami A, Ghoorchian A (2020). A modified carbon paste electrode based on Fe3O4@ multi-walled carbon nanotubes@ polyacrylonitrile nanofibers for determination of imatinib anticancer drug. J. Appl. Electrochem..

[19] Ghanbari K, Roshani M, Goicoechea H, Jalalvand A (2019). Developing an elegant and integrated electrochemical-theoretical approach for detection of DNA damage induced by 4-nonylphenol. Heliyon.

[20] Ghanavati M, Tadayon F, Bagheri H (2020). A novel label-free impedimetric immunosensor for sensitive detection of prostate specific antigen using Au nanoparticles/MWCNTs-graphene quantum dots nanocomposite. Microchem. J..

[21] Yola M, Atar N, Özcan N (2021). A novel electrochemical lung cancer biomarker cytokeratin 19 fragment antigen 21–1 immunosensor based on Si 3 N 4/MoS 2 incorporated MWCNTs and core–shell type magnetic nanoparticles. Nanoscale.

[22] Alnaimi A, Al-Hamry A, Makableh Y, Adiraju A, Kanoun O (2022). Gold nanoparticles-MWCNT based aptasensor for early diagnosis of prostate cancer. Biosensors.

[23] Haghshenas E, Madrakian T, Afkhami A (2015). A novel electrochemical sensor based on magneto Au nanoparticles/carbon paste electrode for voltammetric determination of acetaminophen in real samples. Mater. Sci. Eng., C.

[24] Madrakian T, Haryani R, Ahmadi M, Afkhami A (2016). A sensitive electrochemical sensor for rapid and selective determination of venlafaxine in biological fluids using carbon paste electrode modified with molecularly imprinted polymer-coated magnetite nanoparticles. J. Iran. Chem. Soc..

[25] Aguedo J, Lorencova L, Barath M, Farkas P, Tkac J (2020). Electrochemical impedance spectroscopy on 2d nanomaterial mxene modified interfaces: Application as a characterization and transducing tool. Chemosensors.

[26] Šišoláková I, Hovancová J, Oriňaková R, Oriňak A, Garcia D, Shylenko O, Radoňák J (2019). Comparison of insulin determination on NiNPs/chitosan-MWCNTs and NiONPs/chitosan-MWCNTs modified pencil graphite electrode. Electroanalysis.

[27] Brett C (2022). Electrochemical impedance spectroscopy in the characterisation and application of modified electrodes for electrochemical sensors and biosensors. Molecules.

[28] Uygun Z, Uygun H (2014). A short footnote: Circuit design for faradaic impedimetric sensors and biosensors. Sens. Actuators, B Chem..

[29] Md Azahar A, Mondal K, Jiao Y, Oren S, Xu Z, Sharma A, Dong L (2016). Microfluidic immuno-biochip for detection of breast cancer biomarkers using hierarchical composite of porous graphene and titanium dioxide nanofibers. ACS Appl. Mater. Interfaces..

[30] Chun L, Kim SE, Cho M, Choe WS, Nam J, Lee DW, Lee Y (2013). Electrochemical detection of HER2 using single stranded DNA aptamer modified gold nanoparticles electrode. Sens. Actuators B Chem..

[31] Hezard T, Fajerwerg K, Evrard D, Collière V, Behra P, Gros P (2012). Influence of the gold nanoparticles electrodeposition method on Hg (II) trace electrochemical detection. Electrochim. Acta.

[32] Lim S, Lim H, Joo Y, Jeon D (2020). Impact of MWCNT concentration on the piezo-impedance response of porous MWCNT/PDMS composites. Sens. Actuators A Phys..

[33] Frei M, Aradhya S, Hybertsen M, Venkataraman L (2012). Linker dependent bond rupture force measurements in single-molecule junctions. J. Am. Chem. Soc..

[34] Xia Y (2020). Kinetic analysis of the reduction processes of a cisplatin pt (iv) prodrug by mesna, thioglycolic acid, and thiolactic acid. J. Chem..

[35] Gatabi M, Milani Moghaddam H, Ghorbani M (2016). Point of zero charge of maghemite decorated multiwalled carbon nanotubes fabricated by chemical precipitation method. J. Mol. Liq..

[36] Crespo P, Litrán R, Rojas T, Multigner M, De la Fuente J, Sánchez-López J, García M, Hernando A, Penadés S, Fernández A (2004). Permanent magnetism, magnetic anisotropy, and hysteresis of thiol-capped gold nanoparticles. Phys. Rev. Lett..

[37] Afkhami A, Kafrashi F, Ahmadi M, Madrakian T (2015). A new chiral electrochemical sensor for the enantioselective recognition of naproxen enantiomers using l-cysteine self-assembled over gold nanoparticles on a gold electrode. RSC Adv..

[38] James CA, Mant TG, Rogers HJ (1987). Pharmacokinetics of intravenous and oral sodium 2-mercaptoethane sulphonate (mesna) in normal subjects. Br. J. Clin. Pharmacol..

[39] Ma J, Shi J, Le H, Cho R, Huang J, Miao S, Wong B (2008). A fully automated plasma protein precipitation sample preparation method for LC–MS/MS bioanalysis. J. Chromatogr. B.

